# Increasing Physical Activity via Provider Support and Engagement Using a Digital Health Platform in Adults With Multiple Sclerosis: Protocol for a Randomized Controlled Trial

**DOI:** 10.2196/72213

**Published:** 2025-11-21

**Authors:** Dawn M Ehde, Sarah B Simmons, Kevin N Alschuler, Tracy E Herring, Andrew T Humbert, Susan R Robles, Otari Ioseliani, Karla Landis, Laurie B Kavanagh, Cindy Y Lin

**Affiliations:** 1 Department of Rehabilitation Medicine University of Washington Seattle, WA United States; 2 Ubicomp Lab Department of Computer Science & Engineering University of Washington Seattle, WA United States; 3 Department of Rehabilitation Medicine The Sports Institute University of Washington Seattle, WA United States

**Keywords:** multiple sclerosis, exercise, physical activity, digital health, mobile app, step count, clinical trial

## Abstract

**Background:**

The benefits of physical activity are well established in people with multiple sclerosis (MS); yet, most people with MS are insufficiently active. Although many apps and devices are available to promote physical activity, these are not connected to electronic health records (EHRs), making it difficult for health care providers to prescribe and monitor their patients’ physical activity. The ExerciseRx platform is an innovative cloud-based, Health Insurance Portability and Accountability Act–compliant software platform (app+provider dashboard) that was developed to bridge the gap between consumer activity tracking devices, such as personal smartphones, and the EHR. The ExerciseRx app tracks a patient’s physical activity using their existing personal smartphone and provides a personalized graded progression in step count goals to increase step count gradually and safely over time. The ExerciseRx app also translates the activity data into actionable metrics on a provider dashboard within the EHR that the provider can use to make activity recommendations and monitor patients’ progress; they can also support patients by providing semiautomated weekly feedback and encouragement in meeting physical activity goals.

**Objective:**

This paper describes the protocol for a randomized controlled trial designed to understand whether the ExerciseRx digital health platform improves physical activity, symptoms, and functioning in adults with MS relative to a waitlist, usual care control.

**Methods:**

Participants are ambulatory adults with MS (n=106) who engage in <150 minutes per week of moderate to vigorous intense physical activity. Enrolled participants are assigned to use the ExerciseRx app for 12 weeks, versus a waitlist, usual care control. Participants allocated to the intervention condition have access to the ExerciseRx app, and their providers have access to the participants’ activity data via a provider dashboard, connected to the EHR. Participants allocated to usual care receive the care they would normally obtain at the MS Center, including encouragement to participate in and increase physical activity as tolerated from their clinical provider and a handout that describes the current physical activity recommendations for adults with MS and links to local and web-based resources suitable for MS. The primary outcome is the change in average daily step count throughout the 12-week intervention. Secondary outcomes include symptoms (fatigue intensity, pain intensity, sleep, and depressive symptoms), patient-reported functional outcomes (physical functioning, fatigue interference, pain interference, falls, and social participation), and qualitative analysis of participant and provider interviews on usability and acceptability of the ExerciseRx platform.

**Results:**

The study was funded in October 2023. Participant enrollment began in March 2024 and will continue through December 2025. As of July 25, 2025, a total of 85 participants have been enrolled in the trial. Data analysis and dissemination preparations will begin in January 2026.

**Conclusions:**

Results of this trial will provide important new information on the efficacy of an innovative digital health intervention tool for physical activity promotion.

**Trial Registration:**

ClinicalTrials.gov NCT06270641; https://clinicaltrials.gov/study/NCT06270641

**International Registered Report Identifier (IRRID):**

DERR1-10.2196/72213

## Introduction

Physical activity promotion is one of the most effective rehabilitation strategies for reducing multiple sclerosis (MS) symptoms and restoring health and functioning. Physical activity improves mobility, balance, fatigue, and depressive symptoms in people with MS [1-4]. Emerging evidence indicates that exercise also improves cognition, including attention, learning, memory, and executive functioning [5]. In addition to its importance in MS symptom management and overall health, regular physical activity may have disease-modifying effects [3,6]. Premorbid physical activity can predict disability progression over 2 years in people with MS [7], and moderate to vigorous levels of physical activity are associated with more normalized gray matter, white matter, and hippocampal volumes in people with MS [8]. Furthermore, a recent randomized clinical trial found that 24 weeks of progressive aerobic exercise resulted in lower MS relapse rates compared to waitlist controls [9].

Although physical activity is a stated priority for people with MS [10,11], approximately 80% of adults with MS do not get sufficient physical activity [12,13]. They are less active than the general US population [14], which is also sedentary [15]. For example, in a 2018 meta-analysis pooling 1355 adults with MS from 10 studies, adults with MS took significantly fewer steps per day relative to a nationally representative sample of 3752 adults [16]. Physical activity also decreases over time as the disease progresses [17] and is low even in those with mild disease or disability [12,14]. Barriers to exercise in this population include the physical effects of MS (eg, mobility impairment, imbalance, and weakness), common MS symptoms (eg, fatigue, depression, and pain), and psychosocial factors (eg, low exercise self-efficacy) [3,18]. Although physical activity is safe for people with MS [19], fears about its safety, potential to exacerbate MS disease, or falls may also present barriers [18]. The lack of community exercise facilities, the costs of gym memberships or wearables, transportation, and time restrictions are also barriers [18].

The National MS Society convened a panel of clinical and scientific experts to develop consensus recommendations for promoting exercise and lifestyle physical activity for people with MS across disability levels. Their 2022 recommendations included that: (1) health care providers (hereafter referred to as “providers”) routinely assess and promote the benefits of and engagement in exercise and physical activity for every patient with MS; (2) providers encourage >150 minutes per week of exercise or >150 minutes per week of lifestyle physical activity; and (3) exercise and physical activity should be advanced gradually, based on the individual’s preferences, abilities, and safety [18]. The guidelines emphasize the essential role of providers in promoting physical activity and encourage providers to support patients with strategies that increase physical activity, such as self-monitoring, goal setting, and accountability [16,18].

Despite a 2012 expert consensus panel recommending that providers incorporate physical activity promotion into comprehensive MS care [20], most people with MS report that their providers rarely discuss physical activity [10,21]. Importantly, adults with MS who met physical activity guidelines reported having providers who promoted physical activity, including through education and specific activity prescriptions, pointing to the central role that providers have in physical activity promotion [21,22]. Adults with MS prefer obtaining activity promotion information directly from their MS providers through patient-provider interactions. They are less interested in passive methods, such as receiving print (eg, handouts and pamphlets) or electronic information (eg, email and web-based) from their providers, although they appreciate having such information to supplement recall after the health care visit [23]. People with MS want their providers to give them up-to-date information about the benefits of activity and clear recommendations tailored to their disease severity, symptoms, and current activity level [10,22]. They also seek behavioral strategies to help incorporate physical activity into their daily lives, including goal-setting tools, accountability measures, and activity trackers, particularly through mobile technology [22,23]. They want these tools to be affordable, convenient, and accessible [22,23].

Research indicates that MS providers also want to support physical activity engagement in their patients with MS [21,24]. Providers recognize the opportunity for patient-provider interactions to discuss and promote physical activity [21,25]. They want clear protocols for prescribing physical activity, including for providing brief advice, discussing the benefits of physical activity, and assessing physical activity behavior [21,25]. They also want tools to help their patients initiate, track, and sustain physical activity and ways to monitor and guide activity [21].

**Figure 1:**
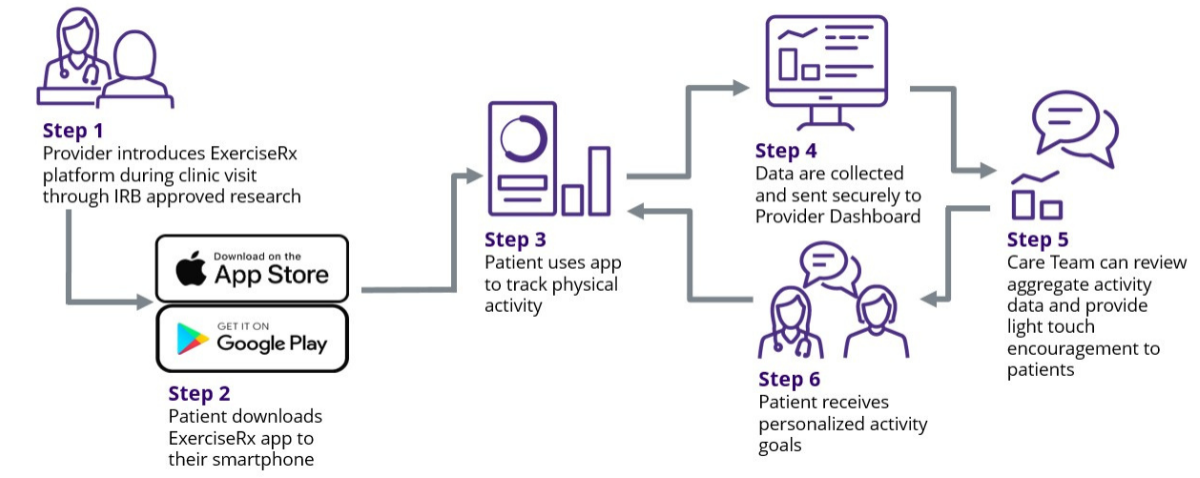
Overview of the ExerciseRx platform.

**Figure 2:**
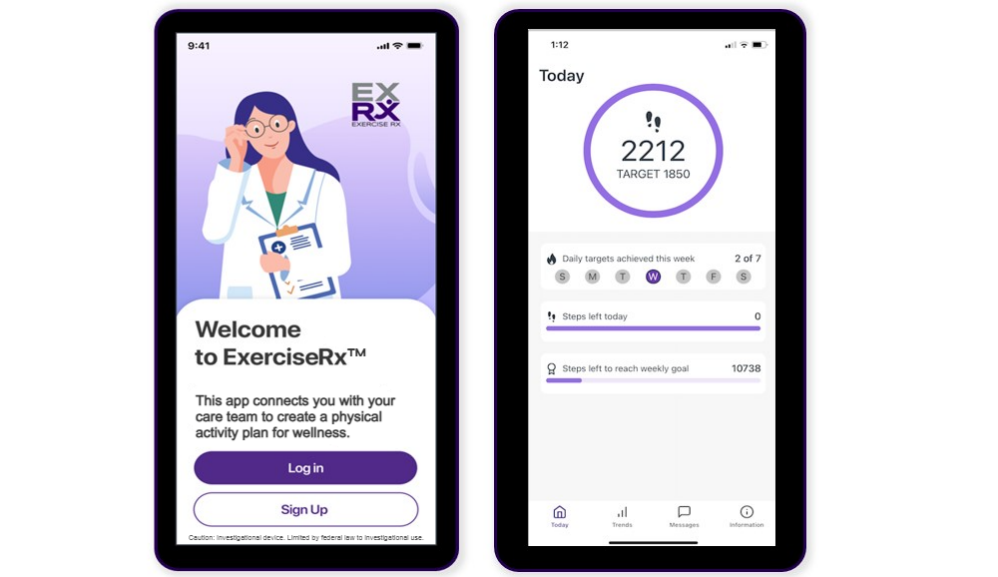
The ExerciseRx app example screenshots.

The ExerciseRx app is an innovative cloud-based, Health Insurance Portability and Accountability Act (HIPAA)–compliant software platform that addresses the current gaps in physical activity support in clinical care. The ExerciseRx app was developed at the University of Washington to bridge the gap between consumer activity tracking devices, such as personal smartphones, and the electronic health record (EHR), thus making physical activity support possible in the clinical setting. Currently available apps and devices (eg, Fitbits) are not connected to EHR systems, making it difficult for health care providers to prescribe and monitor their patients’ physical activity. In contrast, the ExerciseRx app translates summative physical activity data collected by the patient’s existing personal smart devices (Android or iOS phone or tablet) into actionable metrics on a provider dashboard within the EHR (Figures 1 and 2). Each patient is provided a personalized recommendation to facilitate an increased step count gradually and safely over time. Providers can also recommend physical activity tailored to patients’ ability levels and preferences. The platform incorporates behavior change strategies, including motivational feedback and nudges. Through its connection to the EHR, it also facilitates a feedback loop with the provider, fostering caring patient-provider connections around activity promotion and behavior change. The ExerciseRx app leverages patients’ personal smartphone capabilities to track, quantify, and tailor personalized physical activity recommendations, which makes it readily scalable, accessible, and affordable across diverse patient populations and clinical settings.

This paper describes the rationale, aims, and protocol for the ExerciseRx-MS Trial, a randomized controlled trial designed to answer whether the ExerciseRx platform improves physical activity and other common symptoms and functional outcomes in adults with MS who are insufficiently active. The first aim is to evaluate the efficacy of the ExerciseRx platform relative to usual care in increasing physical activity, measured by average daily step count (primary outcome), activity volume, and the proportion who meet exercise guidelines in adults with MS who are insufficiently active. We hypothesize that participants randomized to the ExerciseRx app group will show a greater increase in physical activity at 12 weeks, as measured by daily average step count (primary outcome) and by total metabolic equivalent minutes of activity per week (secondary outcome) relative to participants randomized to usual care. We also hypothesize that a higher proportion of participants in the ExerciseRx app group will increase their minutes per week of moderate to vigorous intensity aerobic activity at 12 weeks (secondary outcome), relative to usual care participants.

The second aim is to evaluate the efficacy of the ExerciseRx platform relative to usual care on secondary outcomes, specifically symptoms (fatigue intensity, pain intensity, sleep, and depressive symptoms) and patient-reported functional outcomes (physical functioning, fatigue interference, pain interference, falls, and social participation). We hypothesize that participants in the ExerciseRx platform group will show greater improvements in secondary outcomes at 12 weeks relative to usual care participants. Additional secondary analyses will test maintenance of any treatment effects at a 3-month follow-up. We will also explore moderators of immediate (post-12 weeks) and 3-month follow-up outcomes, including demographics, disease course, and baseline participant factors (including fatigue, pain, depression, activity level, BMI, exercise self-efficacy, and disability) to explore potential moderators of treatment effects that can be evaluated a priori in future effectiveness and mechanistic research.

We also plan to evaluate implementation factors relevant to future multicenter effectiveness trials and large-scale implementation of the ExerciseRx platform in MS centers (Aim 3)*.* Both quantitative (usability ratings of ease of use, interface, satisfaction, and usefulness, and the frequency and engagement with the ExerciseRx app) and qualitative data collected from both patients and providers will assess these implementation outcomes.

## Methods

### Design

**Figure 3:**
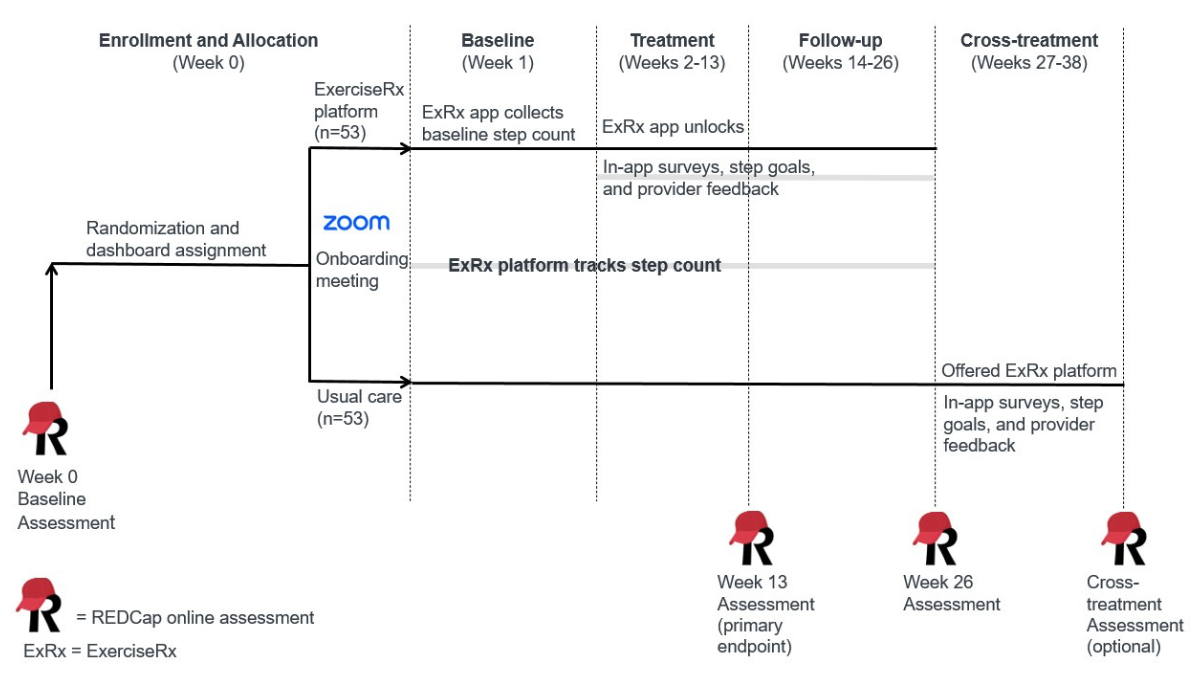
Overview of the ExerciseRx-MS study design. MS: multiple sclerosis; REDCap: Research Electronic Data Capture.

To address Aims 1 and 2, we are conducting a two-group parallel (1:1) assessor-masked randomized controlled trial designed to compare the ExerciseRx platform to usual care. We will use qualitative research methods to explore implementation factors (Aim 3). Outcomes are being collected at baseline, post a 12-week treatment period (primary end point), and at a 3-month follow-up after the treatment period. We selected a 3-month follow-up because it is feasible in a 3-year study and sufficient time to see a change in activity behavior. We preregistered the trial at ClinicalTrials.gov (NCT06270641) on February 21, 2024, before enrolling the first participant. Figure 3 provides the study design and participant flow.

### Ethical Considerations

All study procedures were approved by the University of Washington Human Subjects Division (STUDY00018628) on August 30, 2023. Electronic informed consent is obtained from all study volunteers at the time of enrollment; they are informed that they can opt out of participation at any time during the study. Some identifiers (eg, name, mailing address, ZIP code, email address, phone numbers, and medical record number) are collected for the trial. To protect identifiable participant data, access is restricted to authorized study personnel who are trained in confidentiality and data security procedures. All electronic data are stored on secure, encrypted servers with access controls. Identifiers are stored separately from research data, and all data transfers use secure, encrypted methods. Participants will be informed of confidentiality protections in the consent process, and data will be securely destroyed when no longer needed. Participants are compensated US $150 for completion of all study assessments (US $50 for each of the three assessment points).

### Setting

Participants (n=106) are being recruited from the University of Washington Medicine Multiple Sclerosis Center, a comprehensive, interdisciplinary center that provides specialty care (including neurology, rehabilitation medicine, and psychology) for >3000 patients with MS per year. All clinic patients are offered opportunities to participate in clinical research through a coordinated research registry recruitment effort that is a standard element of the clinic’s patient flow.

### Recruitment, Enrollment Procedures, and Eligibility Criteria

The primary recruitment sources are referrals from clinic providers (physiatrists, neurologists, nurse practitioners, nurses, and psychologists) and the research registry. The EHRs of patients who are potentially interested in and eligible for the study are prescreened by a research coordinator to confirm MS diagnosis [26,27]. The coordinator then meets with the potential participant by phone to complete a standardized eligibility safety screening to ascertain eligibility. Staff track the recruitment outcomes for everyone who is screened, including reasons for ineligibility or declination. Basic demographic and MS disease characteristics (MS duration, course, and date of diagnosis) are obtained for those considered eligible who decline to enroll; this deidentified information will be used to compare them to study participants to assess the sample’s generalizability. All consent procedures are via REDCap (Research Electronic Data Capture; Vanderbilt University), an open-source, secure, HIPAA-compliant web-based platform for data capture (including e-consent and outcome survey administration) [28].

Inclusion criteria are (1) provider-confirmed diagnosis of MS; (2) 18 years of age or older; (3) Patient Determined Disease Steps (PDDS) score <3, indicating the potential for some gait disability although typically ambulates without an assistive device; [29-31] (4) insufficiently active, defined as <150 minutes of moderate intensity physical activity per week per the US National Physical Activity Guidelines, assessed using the Physical Activity Vital Sign [26,27,32] during screening; and (5) use of an iPhone with software version iOS14+ or an Android phone with version 5+. Exclusion criteria are (1) recent (past 4 weeks) or planned surgery during the study period which may impact step counts; (2) MS relapse within the last 30 days; (3) plans during the study period which may impact a participant’s daily access to Wi-Fi, which could interfere with server uploads of mobile phone data; (4) >3 falls in the past 6 months; and (5) contraindication to exercise.

During the safety screening, through questions adapted from the Physical Activity Readiness Questionnaire [33], participants are asked to report any recent history of chest pain during exercise, loss of consciousness, health conditions worsened by physical activity, and other contraindications to exercise. Participants are also asked if they have a history of osteoporosis. If yes, the research coordinator obtains an electronically signed HIPAA authorization from the potential participant for further screening to be completed by a study investigator.

### Randomization, Allocation Concealment, and Masking Procedures

Block randomization, stratified by self-reported sex assigned at birth, is done using a web-based treatment arm assignment system with a 1:1 assignment after the one-week baseline. To ensure concealed allocation, an unmasked research coordinator retrieves the treatment assignment at the time of randomization and informs the participant of their assignment. The coordinator also notifies the participant’s provider of their assignment to the ExerciseRx app group so that they know to look for the participant on the provider dashboard of the ExerciseRx platform. The statistician is masked to treatment allocation.

Participants directly enter their patient-reported outcomes data into REDCap, the web-based data capture platform, which minimizes concerns of staff biases interfering with data collection. Participants are informed of equipoise and that the study’s purpose is to determine whether the ExerciseRx platform or usual care is more beneficial.

### ExerciseRx Intervention

#### Overview

The ExerciseRx platform was developed through a collaboration of investigators from the University of Washington Departments of Computer Science and Engineering and Human Centered Design and Engineering, and The Sports Institute at UW Medicine. It was designed to bridge the gap between consumer activity tracking devices, such as personal smartphones, and providers’ EHRs, thus making recommending physical activity possible as part of routine patient care. The ExerciseRx platform is a cloud-based, HIPAA-compliant, free digital platform that translates clinically relevant activity data collected by the patient’s existing personal smart devices (Android or iOS phone or tablet) into actionable metrics on a provider dashboard within the Epic EHR.

The ExerciseRx platform intervention protocol involves the following.

#### Baseline ExerciseRx Platform Onboarding Meeting With Research Coordinator

After completion of informed consent and the baseline measures, the research coordinator meets with the participant by Zoom (Zoom Communications, Inc) to help them download the ExerciseRx app to their smartphone. The coordinator then teaches them how to use the ExerciseRx app and instructs them to always carry their phone throughout the day. The coordinator reviews the ExerciseRx app procedures with the participant so that they know what to expect during the intervention period. The coordinator also educates the patient on how their step count, as tracked by the ExerciseRx app, is a key marker for physical activity. The coordinator reviews the recommended physical activity guidelines for adults with MS and resources for increasing physical activity and emails them a copy.

#### Baseline Step Count Period (1 Week)

Participants are asked to carry their phone throughout the day for 1 week to evaluate their baseline step count with no app interactions, notifications, or provider feedback during the baseline recording period. The baseline step count is used to calculate personalized step count goals.

#### Initial Personalized Daily Step Target

At the end of baseline, intervention participants receive a notification from the ExerciseRx app providing them with a personalized initial daily step target and weekly goal based on their baseline.

#### Exercise Progression and App Features

The ExerciseRx app displays a weekly step count goal, as well as daily step count targets (see Figure 2 for example). If participants meet their daily step targets, they are on track to meet their weekly step count goal. If 100% of the weekly goal is met the week before, the weekly step count goal increases by 3%. If 75%-99% of the goal is met, there is a 2% increase in their goal. If <75% of the weekly step count goal is met, there is no change to the weekly step count goal. The minimum step count is 1000 steps. The maximum goal in the ExerciseRx app is 10K and will not advance beyond that number, but since the step count is based on the patient’s baseline and progress, the patient may not reach 10K steps during the study period, as it is an individualized progression.

Participants use the app daily for 25 consecutive weeks after the baseline week. Weekly, participants complete an in-app survey of their individual barriers or facilitators to activity. During this period, the app displays daily step count targets, weekly step count goals, weekly summaries, a weekly assessment of barriers or facilitators to activity, semiweekly “Did you know” general and MS-specific messages such as “Exercise is medicine—increasing movement is a great way to better manage MS fatigue and may even reduce risk of relapse!” and provider automated feedback on activity progress (eg, thumbs-up reactions, weekly summaries, supportive messages, or encouragement based on whether the participant is near, at, or below their weekly goal).

#### Provider Dashboard and Interactions

A total of 7 MS providers (2 physiatrists, 4 neurologists, and 1 psychologist) are the providers in this study. Each provider can view on the provider dashboard of the ExerciseRx platform: (1) a triaged aggregate patient panel that depicts their patients’ progress in meeting weekly goals; (2) clinical decision support recommendations for next steps (eg, reactions and message suggestions); and (3) individual participant weekly step count and most common barriers or facilitators (obtained from the surveys completed weekly by participants). The providers check and act on the dashboard at least once a week during the study period. They can offer feedback and reinforcement via the provider dashboard of the ExerciseRx platform, which sends the patient messages via app notifications. For example, if a patient is at less than 75% of their goal by the 3rd week, their MS provider can send them a nudge from the provider dashboard.

#### Low Engagement Support Protocol

The app monitors participants’ engagement and sends automated notifications to the provider and research coordinator. For example, if a patient has not opened the app in 5 days, they receive the following support message: Tech support message #1: “Hello, we noticed you haven’t opened the ExerciseRx app recently. Are you having any difficulties with the app or accessing Wi-Fi or cellular connection? Please let us know if there is anything we can assist you with.” The research coordinator monitors the responses and patient inquiries and provides responses or technical assistance if needed. They also connect the patient to the provider to answer questions as needed; providers can also reach out to participants if they wish.

#### Post 12-Week Intervention Period

Participants in the ExerciseRx app group are allowed to continue using the ExerciseRx app and encouraged to continue with physical activity goals, as indicated. Their providers may also continue using the provider dashboard of the ExerciseRx platform with their patients.

#### Provider Training

The senior author, who helped develop the ExerciseRx platform, and the research coordinator trained the providers on the use of the ExerciseRx platform, including their responsibilities. Although the providers are familiar with the 2020 MS Exercise and Physical Activity Recommendations, CYL (ExerciseRx project lead and sports medicine provider) and SBS (MS physiatrist with exercise expertise) reviewed the guidelines with the providers and described National MS Society and other MS-specific resources (eg, website links and handouts videos) available to providers in educating their patients about the benefits of physical activity in MS.

### Usual Care

#### Overview

The American Academy of Neurology has recommended that patients with MS be counseled by their providers on the benefits of exercise and appropriate physical activity [18]. Participants allocated to usual care receive the care they would normally obtain at the MS Center, including encouragement to participate in and increase physical activity as tolerated from their clinical provider (neurologist, physiatrist, or rehabilitation psychologist), but without the use of the ExerciseRx platform. Specific physical activity guidance varies based on the provider but may include guidance on current exercise recommendations for people with MS (dose, duration, and type), known benefits of exercise in people with MS, links with exercise resources, and referrals to physical therapy or physiatry. In addition, at the beginning of the study intervention period, all participants (including usual care) are given a handout that describes the current physical activity recommendations for adults with MS (type, duration, and intensity), the benefits of physical activity, and links to local and web-based resources suitable to MS (eg, free web-based exercise videos, National MS Society exercise or physical activity educational materials, and links to community resources). We selected the usual care control as a comparator because clinical trials methodologists [34] argue that usual care and waitlist controls are appropriate for efficacy trials when the goal is to promote innovation and test potentially efficacious interventions.

#### Usual Care Onboarding Meeting With Research Coordinator

After completion of informed consent and the baseline measures, the research coordinator meets with the participant by Zoom to review the study procedures. Like participants in the ExerciseRx app group, the coordinator reviews the recommended physical activity guidelines for adults with MS and provides print resources for increasing physical activity, identical to what materials the ExerciseRx participants receive.

#### 26-Week Usual Care Period

Usual care participants are instructed to carry their phone throughout the day to allow passive collection of step counts during the study period; however, they will not be able to view or use any of the features of the ExerciseRx app. Participants are not discouraged from engaging in physical activity, and providers are not precluded from providing whatever care they usually would to their patients during the study period.

#### Post Follow-Up Phase

After the final (26-weeks) outcomes are collected, usual care participants are offered the ExerciseRx platform, which is delivered as described above.

### Assessment Procedures and Measures

#### Overview

All participants complete baseline and outcome assessments using REDCap, a web-based data capture platform [28] that can be completed by any digital device with internet access. It includes a human verification service (reCAPTCHA) to avoid responses from bots. Outcome assessments are collected at baseline, post a 12-week treatment period (primary end point), and at a 3-month follow-up after the treatment period. We obtain step count data from all participants throughout the trial and collect data from all randomized participants, regardless of their adherence to treatment (ie, intent-to-treat). Participants receive up to US $150 remuneration for completing the REDCap assessments (US $50 for each assessment point).

#### Participant Characteristics

Participants provide demographic variables, including self-reported sex assigned at birth, gender identity, age, race, ethnicity, employment status, relationship status, and education level. Clinical information, including MS subtype, time since diagnosis, and medications (including disease-modifying therapy), is obtained from chart review. MS disease severity (assessed using the PDDS, a brief self-reported assessment that has been found to be a valid measure of disease severity in an MS population [31]), occurrence of clinical relapses (over the preceding 6 months and throughout the study period), and self-reported falls (over the preceding 6 months and throughout the study period) are also assessed. Data on other treatments and therapies used during the study period (active treatment and follow-up) are collected via medical record extraction and self-report.

#### Primary Outcome: Step Count

For the week before (baseline) and throughout the 12-week intervention period, step count will be measured within the ExerciseRx app, which passively collects step count data from device sensors using Core Motion, Google Fit, and Google Health Connect. Participants in the usual care condition will also have their step count passively monitored via the ExerciseRx app on their smartphones. Step count is an ideal primary outcome in physical activity promotion trials because it provides an objective, quantifiable measure of daily activity that is sensitive to change and free from self-report bias. It captures both structured and incidental movement, is easy to interpret for participants and clinicians, and is associated with meaningful improvements in health and function [35], including in adults with MS [36,37]. It is possible that step count may be underestimated due to a lack of carrying time or location; a few studies have shown that carrying a phone in a pocket or purse can lead to underestimations of step count [38,39]. However, since this study is looking primarily at changes and trends in step count over time, with consistent phone carrying behaviors, clinically relevant assessments will still be obtained [38,40].

#### Secondary Outcomes: Physical Activity

The International Physical Activity Questionnaire-Short Form, which is validated for MS [41,42], will be given to participants at baseline to assess frequency and intensity of physical activity at each assessment timepoint. Activity intensity and frequency will then be used to calculate total MET-minutes per week for participants in each condition. The Physical Activity Vital Sign (described above) will also be obtained at each timepoint to determine the percentage of participants in each group that progress their total minutes spent in moderate to vigorous intensity physical activity per week toward the current physical activity guidelines (of >150 min of moderate intensity or >75 min of vigorous activity per week).

#### Secondary Outcomes: Symptoms and Function

The following patient measures, all validated in MS, are obtained at the three outcome timepoints to investigate the effects of the ExerciseRx platform on symptom severity: Patient-Reported Outcomes Measurement Information System (PROMIS) pain intensity (PROMIS Pain Intensity Short Form) [43,44], fatigue severity (PROMIS Fatigue Intensity Short-Form)[43,45], depressive symptom severity (PROMIS Depression Short-Form) [43,46], and sleep disturbance (PROMIS Sleep Disturbance Short Form) [43,47]. We will also assess functional outcomes, specifically physical functioning (PROMIS Physical Function 10a) [48], pain interference (PROMIS Pain Interference Short-Form) [43,44], fatigue interference (PROMIS Fatigue Impact Short Form) [43,45], social participation (PROMIS Satisfaction with Participation in Social Roles Short Form 8a) [43], and self-reported falls (during the 6 months prior to randomization and throughout the study period).

#### Exploratory Mechanisms: Moderators

We will use baseline values of the demographic, clinical, and symptom measures, exercise self-efficacy [49], and BMI to explore moderators of immediate (post 12 weeks) outcomes that may be evaluated a priori in future effectiveness and mechanism research on the ExerciseRx platform.

#### Implementation Measures

Both quantitative and qualitative implementation measures will be completed by participants in the ExerciseRx app group after they have completed the 12-week treatment period. Quantitative measures will include ratings of ease of use, interface, satisfaction, and usefulness from the mHealth App Usability Questionnaire [50]. Participants’ app engagement will be measured via the ExerciseRx platform data, which tracks the frequency of logins and completion of weekly barriers or facilitators in-app surveys. Providers will also complete a similar brief survey of implementation factors, including measures of acceptability, satisfaction, demand or engagement, usability, and integration of the ExerciseRx platform into their clinic workflows. The ExerciseRx platform will provide data on providers’ frequency of and engagement with the provider dashboard of the ExerciseRx platform, including logins, screen interactions, and messages sent to participants.

In addition to the participant and provider self-report usability measures, we will conduct semistructured interviews with 14 individuals who receive the ExerciseRx intervention and at least 3 providers to better understand their experiences on study completion, including usability and feasibility of the platform. The semistructured interviews are informed by (1) the Lived Informatics Model [51], which describes key stages of using health tracking apps: deciding to track, selecting a tool and tracking plan, collecting data, integrating it, reflecting on it, lapsing, and resuming use; and (2) goal directed self-tracking, which supports examining how the functionality and design of a tracking app aligns with their activity goals [52]. The interview guide is provided in Multimedia Appendix 1. Interviews will include information regarding demographics, open-ended questions about barriers or facilitators to physical activity and use of technology, and app features feedback. Interviews will be conducted over Zoom, recorded, transcribed, and double-coded with two coders using Dedoose software (SocioCultural Research Consultants, LLC).

#### Safety

We are tracking any adverse events, including falls or injuries related to study participation per University of Washington Human Subjects Division procedures, which include monitoring for possible adverse events, managing them, and reporting them to the principal investigator, Human Subjects Division, and study sponsor, as indicated. Since the ExerciseRx app is a wellness platform that helps patients to meet current national physical activity guidelines, the regulatory pathway is Software as a Medical Device Class I (low risk, exempt).

### Sample Size, Power, and Analysis Plan

Assuming a 15% noncompletion, we plan to enroll 106 participants in the trial to ensure 90 participants complete the study. With 90 participants (45 in each group), we will have 80% power to detect a significant difference in the change in mean daily step count from baseline to 12 weeks between the treatment and control groups for a moderate standardized effect size (Cohen *d*=0.60) using a two-sided significance level of .05.

For Aim 1, we will analyze the efficacy of the ExerciseRx platform using a linear mixed effects model with average daily step count each week as our outcome, fixed effects for week, treatment assignment, and the week-treatment interaction, and a random intercept for each participant. The presence of a significant interaction will indicate that the change in step count over the 12-week study period is impacted by treatment assignment. If differences in demographic or other clinically relevant characteristics between the two groups are found in the descriptive analysis, sensitivity analyses will be conducted, including those characteristics as adjustment variables in the model. Post hoc analyses will include independent and paired sample 2-tailed *t* tests to determine if there are differences between treatment groups at specific time intervals or differences between specific time intervals within a treatment group.

Similar models will be used for Aim 2 and for the secondary physical activity outcomes (activity volume and proportion meeting physical activity guidelines), using each of the secondary outcome measures as the outcome variable in the linear mixed-effects model. As no correction for multiple comparisons will be implemented for these analyses, any significant findings will be considered as hypothesis-generating.

For the qualitative analysis, the interview transcripts will be coded using Dedoose software. Two trained qualitative analysts will independently analyze the data using reflexive thematic analysis, a well-established method for interpreting qualitative data [53]. This approach emphasizes the researchers’ active role in identifying patterns across the data and involves intentional engagement with subjectivity and reflexivity throughout the analytic process. In conducting our analysis, we will draw upon our diverse backgrounds in design, health informatics, patient care, and clinical research to interpret the data and generate themes, while remaining attentive to how participants’ experiences and attitudes may differ from our initial assumptions.

## Results

The study was funded in October 2023. Participant enrollment began in March 2024 and will continue through December 2025. As of July 25, 2025, we have enrolled 85 participants. Data analysis and dissemination preparations will begin in January 2026, and results of the ExerciseRx-MS trial are expected to be submitted for publication by September 2026.

## Discussion

### Principal Findings

Physical activity promotion has emerged as a potent, restorative rehabilitation strategy. In this clinical trial, we aim to advance the scientific understanding and potential future implementation of the ExerciseRx app, a digital health platform for increasing activity in insufficiently active adults with MS. The study leverages the benefits of physical activity, the identified preferences of patients and providers, and a unique, innovative tool for addressing gaps in the translation of activity recommendations into the MS community. The ExerciseRx platform is unique in that it not only addresses several of the barriers to physical activity (eg, convenience and tailored activity recommendations) identified in MS, but it also integrates the MS provider into activity promotion. It ensures that patients are getting the information and guidance they want from their providers [54] and gives providers insight into their patients’ activity levels while minimizing the provider’s clinical burden (through automated features). The trial will also explore the implementability of the ExerciseRx platform to understand contextual factors, which will inform implementation strategies for the next stage of research and accelerate future implementation if the intervention is efficacious.

The ExerciseRx platform can be universally applicable, both to the general population and to a wide range of rehabilitation populations with physical activity needs. Given the focus of our research, as well as the unique needs previously identified in the MS population, this trial is focused on the ExerciseRx platform’s application to the MS population. The ExerciseRx platform has the potential to provide what patients with MS want: clear activity recommendations from their provider and accessible, cost-effective tools for goal setting, accountability, and activity tracking [22,23]. Research has shown that support and guidance from providers [55]—as well as behavioral factors such as self-monitoring of physical activity, setting appropriate goals, and having someone to be accountable to—facilitate physical activity [18]. The ExerciseRx platform also offers what MS providers want: clinically integrated, streamlined tools to help their patients initiate, track, and sustain physical activity. It also helps them guide their patients’ activity without adding to the clinical care burden [21].

### Study Limitations

Inclusion or exclusion criteria were selected to maximize generalizability, and the study was designed to leverage participants’ own technology (ie, smartphones) to reduce barriers to implementation. Unfortunately, current smartphone software is less robust at tracking physical activity in people who are nonambulatory or who use an assistive device for ambulation. In addition, the 2020 Physical Activity Guidelines for people with MS suggest that referrals to specialists (to assure proper safety, form, and appropriate intensity) are more essential in individuals with higher disability [18]. Therefore, the criterion requiring a PDDS of ≤3 (indicating MS may interfere with gait but typically ambulates independently) was selected. We recognize that this range limits the generalizability of the sample to those who can walk and have milder disability, which we decided was acceptable for this stage of research, given that we are interested in the feasibility of the ExerciseRx platform in MS clinical care and wanted to maximize safety (reduce fall risk). If the ExerciseRx platform is found to be efficacious in ambulatory adults with MS, a future study could integrate other features of the ExerciseRx app that are now available into a future clinical trial, including the ability to prescribe a home exercise program incorporating strength training, seated exercises, and other types of physical activity applicable to a broader range of disability levels and activity types in MS. Requiring patients to have a smartphone might also exclude some people from participating. However, recent research on smartphone use in the general population [56] and in MS samples [57] indicates that 85%-90% own a smartphone.

Step count provides a pragmatic, scalable, and accessible outcome well-suited for real-world applications. However, it is possible that there will be variation in step count data across participants in the intervention and control groups. We have several procedures for mitigating this potential limitation. At the onboarding visit, the research coordinator discusses with all study participants the importance of carrying their phones throughout the study and the rationale for it (to consistently capture step count data). They are instructed to carry their phone all day while awake. She also discusses with each participant their phone-carrying habits and helps troubleshoot any potential issues. At the 13- and 26-week assessments, participants are asked to estimate what percentage of their waking hours they carried their phone as a measure of adherence to phone carrying; we will use this measure to explore patterns of self-reported phone carrying, including differential patterns between the two conditions.

### Conclusions

The ExerciseRx-MS trial tests an innovative patient-provider digital health platform, which is designed to increase physical activity using the individual’s own technology (ie, smartphones). The ExerciseRx platform connects adults living with MS and their care providers, who can monitor progress in step count goals and provide encouragement in a minimally burdensome fashion. This trial will evaluate both the efficacy of the ExerciseRx platform in increasing physical activity and the implementability of the ExerciseRx platform in clinical practice, exploring critical contextual factors, including participants’ and providers’ perspectives.

## Appendix

Multimedia Appendix 1 Poststudy interview guide.

Multimedia Appendix 2 Peer review report from Accelerating Functional Recovery of Multiple Sclerosis Grant Committee, National Multiple Sclerosis Society (USA).

## Abbreviations

EHR: electronic health record
HIPAA: Health Insurance Portability and Accountability Act
MS: multiple sclerosis
PDDS: Patient Determined Disease Steps
PROMIS: Patient-Reported Outcomes Measurement Information System
REDCap: Research Electronic Data Capture

## Data Availability

The datasets generated and analyzed during this study are not publicly available due to ongoing data collection, but a deidentified version of aggregated trial data will be available from the corresponding author upon reasonable request following the completion of the study.
