# Supplementary material for: Increasing Physical Activity via Provider Support and Engagement Using a Digital Health Platform in Adults With Multiple Sclerosis: Protocol for a Randomized Controlled Trial
Source: JMIR Res Protoc. 2025 Nov 21;14:e72213. doi: 10.2196/72213 (PMC12680936; doi:10.2196/72213)
Supplement: Multimedia Appendix 1 [file resprot_v14i1e72213_app1.docx]

[Introduction to the Session 1](#_Toc722798065)

[Interview Questions 2](#_Toc779730669)

[General Experience with App 2](#_Toc675527876)

[Step Count Goal 3](#_Toc1266062029)

[Phone Carrying Habits 3](#_Toc1604356153)

[Cycle of Engagement 3](#_Toc958302483)

[Making Sense of Data 4](#_Toc953346448)

[App Features (Notifications, Messages) 4](#_Toc1095166683)

[Clinic Connection Impact 4](#_Toc983799664)

[Resources 4](#_Toc743090911)

[Conclusion 5](#_Toc1105643842)

*Focus of the interview: open-ended questions about barriers/facilitators to physical activity and use of technology, and app features feedback*

Interviewer instructions:

- When you encounter a question that has parts labeled a or b below it, follow these two steps:
  - First, ask the main question
  - Then proceed to ask the lettered follow-up question

General Probes to ask throughout the interview. Try to avoid Yes or No questions and follow short answers with leading(?) questions to encourage elaboration of details

- I’m not sure I understand ________. *or* I’m not familiar with _________. Would you clarify what that means to make sure I understand.
- Can you give me an example of __________? (when do not understand or want them to elaborate or want them to generate something more concrete)
- Tell me more about _______?
- What does that look like for you?
- Let me make sure I am hearing you right [summarize], you’re telling me [this, this and this], did I get that right? Is there anything else you would add?

# Introduction to the Session

- Thank you for taking the time to speak with me today.
- I’m [name] and I am a research coordinator for the study.
- Primarily, I’ll be reading from a script to keep the interviews consistent.
- The interview will take between 30-45 minutes.
- As a reminder, you will receive $50 as a thank you for participating in this interview. A check will be mailed within a few weeks, the same way you were paid for the study surveys. Have there been any changes to your mailing address in the last 3 months?
- The purpose of this conversation is to better understand your experience with the ExerciseRx app and hear your feedback about the app’s design.
- We’re interested in hearing what worked and what did not work well for you. This will help us to improve the app for future patients.
- At the end of the interview, I will ask if you would be willing to provide any quotes about your experience in the study for our research team’s use in publications, social media, and future grant applications. We would only list your quote as coming from your age and gender. Example: *quote* - Male, 57.
- Your participation in this conversation is completely voluntary. You may stop the interview or skip questions at any time. I’m also happy to answer any questions you may have.
- Do you have any questions before we start?
- Now, I’d like your permission to audio-record our conversation. We will remove anything that might identify you from the recording.
  - **Do I have your permission to audio-record our conversation today?**
    - *[If yes, proceed. If no, thank person for their time and let them know you’ll end the Zoom. Ask if they have any questions, and either respond to them or let them know you’ll end the Zoom. Thank them for their time and end Zoom.]*

# Interview Questions

## General Experience with App

The next couple of questions are about your general experience with the App.

[[Screenshare screenshots](https://uwnetid.sharepoint.com/:p:/r/sites/the_sports_institute/Shared%20Documents/The%20Sports%20Institute/Initiatives/ExerciseRx/Research/ExerciseRx%20-%20Multiple%20Sclerosis/Post-Study%20Interviews/MS%20Study%20Post%20Interview%20_Screenshots.pptx?d=w4429bfd741fe4ef8abf82207af9d5c61&csf=1&web=1&e=5pn7Gc) -- slide 1 only at this time -- if ppt wants to refresh their memory about what the app looks like.]

1. Tell me about your experiences using the ExerciseRx app. What stood out as positive? What stood out as negative?
2. On the ExerciseRx app, you were prompted to fill out a weekly survey of the barriers and facilitators for meeting your step count goal. How was this process for you?

## Step Count Goal

The next few questions are about your step count goal.

1. Tell me how you thought about your step count goal as part of your daily and weekly routine?
2. How would you describe the difficulty of the step count goals the app set for you?
   1. How did the difficulty of your step count goal change over the course of the study?

## Phone Carrying Habits

Because we rely on cell phones to track your steps and deliver ExerciseRx, the next questions are about your phone carrying habits.

1. Did your phone-carrying habits change at all because of participating in this study? If so, how did they change?
   1. Do you think that your step count was accurately recorded by the app? Why or why not?
2. What helped and what got in the way of carrying your phone throughout the day? How did you typically carry your phone – in your hand, clothing pockets, a bag, or a purse?
3. Would you have preferred a wearable such as a Fitbit or Apple Watch instead? Why or why not?

## Cycle of Engagement

1. Throughout the study, were you able to increase your number of steps through the personalized goals on the app?
   1. If so, how did you do it? Where (inside or outside) did you walk more? What specific strategies helped you to walk more during the day?
2. How often did you find yourself opening the app and what information were you most often seeking?
3. If your MS provider asked you to use the app long-term, how long do you think you would use the app before stopping or taking a break? (In weeks? Months?)
4. Is there a certain long-term goal you would want to achieve through the app?
   1. *Examples:* For some people, this might be achieving a certain energy level throughout the day, establishing a habit of a certain amount of physical activity each day or week, or being able to walk a certain distance without feeling tired.
5. Are there any specific goals related to your MS that you hoped to achieve by using the ExerciseRx app?
   1. E*xamples:* influencing fatigue/energy, spasticity/spasms, pain levels, or resulting in better overall brain health.
6. What could we change about the app design or features that would make you likely to use the app longer?
7. What features of the app were most helpful in supporting you?
8. Have you noticed a change in your physical activity since using the app?

## App Features (Notifications, Messages)

Next, we are going to ask about the various app notifications and messages.

1. Which were the most motivating for you to hit your step count goals? Which were the least motivating or helpful?
   1. Goal updates
   2. Login reminders
   3. DYK’s
2. How did you feel about the timing and frequency of the push notifications?
3. What changes, if any, would you like to see made to the messages? Do you have any suggestions on messages that we should add for people with MS?

## Making Sense of Data

1. How did the app make it easier to understand your physical activity data?
   1. How could the app better help you make sense of changes or trends in your data? (i.e. graphs, different visual representation)
2. Did you encounter any difficulties or confusion in interpreting the information provided? If so, please describe.

## Clinic Connection Impact

Participating providers have access to a dashboard that allows them to see their patients’ step count data and send them feedback messages.

1. Was the doctor who reviewed your step count throughout the study also one of your MS providers? (yes or no)
   1. [IF YES TO QUESTION 1] How did the app’s connection to your provider influence your experience with it?
      1. Could this connection via the app be improved?
      2. How did you feel about them reviewing your weekly step count?
      3. How did you feel about your provider seeing that you didn’t meet your step count goal?
   2. [IF YES TO QUESTION 1] What other things could your provider do to support you in being physically active?
   3. [IF NO TO QUESTION 1] Do you believe your experience with the app would have been different if one of your doctors reviewed your step count? Why or why not?
2. Do you currently use MyChart?
   1. If yes, how would you feel about using ExerciseRx as part of MyChart?
   2. Would you prefer to have ExerciseRx as a standalone app separate from MyChart?

## Resources

1. At the beginning of the study, we shared an investigator-curated exercise guidelines handout with you. Were these helpful? Why or why not?
2. What other types of resources or tools paired with the ExerciseRx app would have helped you to better achieve your physical activity goals?
   1. *Examples:* handouts on spasticity management, how to deal with heat, exercise videos/demonstrations, or more info on benefits of exercise for people with MS.

## Conclusion

- Before we wrap up, is there anything else you’d like to share about your experience using ExerciseRx?
- Thank you so much for participating!
