# Supplementary material for: Increasing Physical Activity via Provider Support and Engagement Using a Digital Health Platform in Adults With Multiple Sclerosis: Protocol for a Randomized Controlled Trial
Source: JMIR Res Protoc. 2025 Nov 21;14:e72213. doi: 10.2196/72213 (PMC12680936; doi:10.2196/72213)
Supplement: Multimedia Appendix 2 [file resprot_v14i1e72213_app2.pdf]

National Multiple Sclerosis Society

Application Details

**Funding Opportunity:**	Accelerating Functional Recovery of Multiple Sclerosis
**Program Officer:**	Anna Lampe
**Grant Administrator:**	Phyllis Motta
**Application ID:**	RFA-2212-40550
**Principal Investigator:**	Dawn Ehde
**Organization Name:**	University of Washington
**Project Title:**	Increasing Physical Activity via Provider Prescription and Engagement: Efficacy of Exercise Rx for Adults with Multiple Sclerosis

▼ Reviewer Comments

Below are the reviewers' comments concerning your application. These are provided to assist you in understanding the outcome of the review and in preparing a resubmission, should you decide to do so.

Reviewer 1

- Consider the feasibility of clinicians having enough time to provide feedback and timely interaction to make the program actionable, especially if the patient only sees MD and PT instead of PM&R or physiatry. Is one training timepoint enough for training the clinician?
- Evaluate the rationale for not including a physical therapist on the team, particularly in regards to evaluating gait, balance, safety, and aiding in exercise prescription.
- Reconsider the frequency of weekly questionnaires, as previous similar studies in MS have found this to be burdensome for patients with MS – or provide justification for this timing schedule.
- Consider using PR-EDSS instead of PDDS if the goal is to follow EDSS, as it is a validated and same-scale alternative.
- Explore the possibility of including wearable watches/on wrist or finger to improve carry time, in addition to smartphones.
- Re-evaluate the use of "step count" as a proxy for physical activity, and provide further information on the validity of this measurement.
- Include outcomes validity and reliability with MCID/MDC for primary outcomes – and secondary if available.
- Provide a stronger rationale for the percentage increase in STEPS, such as why a 3% increase is chosen for 100% of goal attainment (pg 8)

Reviewer 2

Thank you for this novel work and proposal

- Excellent to address wider implementation feasibility of app integrated with electronic health record
- Strong collaborative team developed app in partnership with patients and providers= very good: "scientific team who developed ExerciseRx - The Sports Institute at UW Medicine, UW Computer Science & Engineering Ubicomp Lab, and UW Human Centered Design & Engineering (led by Cindy Lin, MD) - and experts from the UW Multiple Sclerosis Rehabilitation and Wellness Research Center (led by Dawn Ehde, PhD, and Kevin Alschuler, PhD…" And Team has published together previously.
- Extremely well written proposal with solid background/literature review and rational why we need health care providers to prescribe exercise.
- Like that "ExerciseRx's unique algorithm addresses this by transforming a patient's baseline physical activity profile into an individualized, safe plan for gradually increasing physical activity and associated behavior changes.
- Like Pilot data presented with n=26 with a 68% responder rate for # who increased their step counts. Mean statistically sig increase in average step counts.
- Good recruitment focuses on people not physically active via screening with the Physical Activity Vital Signs Score, nicely explained rational why not recruiting higher PDDS/EDS scores to follow current 2020 PA guidelines -- - however might consider doing that anyway as this is the population who are most likely to benefit from this type of intervention with min risk.

Weaknesses

- prescribers are very busy clinicians-- can their work prescribing and monitoring be translated /estimated into minutes of work/week rather than a simple frequency count? The data would be useful in terms of later implementation-see below.
- aim #3 is more critical than aim #2 as a substantial amount of literature already exists for what PA does for MS symptoms. What you really want to know is does the app increase PA behavior to meet PA guidelines and will it be feasible for wider implementation? Suggest do not burden patients or team with excessive data collection that is less likely to change practice.
- while the app is great -- evidence suggests that the prescriber themselves, and whether they are viewed as an expert in what they do from the perspective of the patient matters with respect to adherence, the treatment response (and the placebo response). This is true for most if not all medical interventions, including exercise . For the brief exit surveys from prescribers – collect data on their confidence levels with prescribing PA/using the app/their self-rated knowledge expertise about PA prescription, their years of experience in MS care, professional background etc.
- Can you recruit any providers from a non-MS specialty practice to participate in this study? This would strengthen the external validity.
- Describe how long the orientation training will take with the prescribers and if this will be tracked? Will they be able to get help/guidance after the training?: From the Grant: "The investigators will meet with the MS Center providers (neurologists, physiatrists, nurse practitioners, and psychologists) to review the study protocol. Dr. Lin will train the providers on the use of the ExerciseRx platform, including their responsibilities. Although the providers are familiar with the 2020 MS Exercise and Physical Activity Recommendations, Drs. Lin (ExerciseRx expert) and Simmons (MS physiatrist) will review the guidelines with the providers and describe National MS Society and other MS-specific resources…"
- Buy in from prescribers is possibly the greatest implementation barrier your team will face taking this outside your center or possibly even within. Suggest prepare for these challenges well. From the Grant "During the study period, the providers will be asked to check and act on the dashboard at least once a week." Suggest include more than 3 prescribers/providers in the exit interviews --- do exit interviews with them until you have data saturation, track if they actually check the dashboard once a week?

This is a great study - I was excited to read your proposal. It made me want to imagine a future where every EMR may have a "health behavior prescription" section with individual health behavior vital signs (will need associated compensation on the provider side for the time and expertise it takes to prescribe). Suggest that you get as much feedback as you can to inform your research direction and help with testing implementation. Suggest that you get an MS neurologist on the proposal -may strengthen the proposal, add also a patient partner.

Other

The proposal tests an EHR-integrated platform for promoting physical activity among adults with MS. The platform leverages patients' phones to track, quantify, and tailor personalized physical activity solutions, enabling healthcare providers to prescribe and monitor physical activity as part of routine care. The authors plan to conduct a randomized controlled trial with 106 insufficiently active adults with MS to evaluate the efficacy of ExerciseRx in increasing physical activity and improving common symptoms and functional outcomes.

Comments on the relevance to the RFA:

The proposal addresses the RFA's focus on developing novel non-pharmacological approaches for people with MS by leveraging technology to promote physical activity, which has the potential to improve physical function in MS patients.

Comments on the study design and analysis:

(1) The proposed analyses are well-described, though I am curious whether the authors would define the treatment fixed effect or the treatment*time interaction as primary. I can see arguments for both, but at the end of the day they will capture different dynamics. Given the lengthened timespan of the study - relative to the pilot study - it is also perhaps worth designing the analysis to detect and account for potential non-linearities in the effect (e.g., a plateau after 9 weeks).

(2) Relatedly, the study is powered based on a continued (if slightly attenuated) trend from the 8-week pilot study. If there is a plateau, the study might be slightly underpowered to detect what could still be a clinically meaningful effect.

(3) The authors seem highly capable of running a clean study with few dropouts, but it would be important to see a missing data plan regardless. Specifically, the authors mention assuming up to 15+% non-completion and design recruitment such that they can perform analysis on only the completers. That does not seem suitable for this design - if there is differential completion between the intervention and control groups (which is plausible, especially since the control group data seems to be primarily passively collected), or if non-responders are more likely to drop out of the intervention group (also plausible), then the estimate of the effect could be biased. This is especially true when studying the temporal slope. So a detailed plan for accounting for missing data would be important.

Community Review

The Community Review of MS Research Committee expressed enthusiasm about this project. They appreciate that physical activity is important to the quality of life of those living with MS and that many people living with MS are not currently getting enough physical activity. However, the committee did express concerns about the inclusive nature of the study design and the ability of the project to be implemented broadly.

- Overall, there was strong enthusiasm for the focus of this study.
- The committee appreciated that this project would open up a dialogue between healthcare providers and people living with MS about the importance of physical activity and that a concrete plan will be created that is tailored to the individual.
- The committee appreciated that this study focused on a non-pharmaceutical intervention.
- The committee was concerned that they and their neurologist would not have enough time during and between their regular appointments to fully review the data and tailor the exercise prescription to the individual. It may be helpful to clarify which members of the care team will be responsible for reviewing physical activity data and coordinating the tailoring of the program.
- There was concern that the use of smartphones and the requirement for internet and data access may exclude some members of the MS committee from participating.
- The committee also expressed disappointment that those living with MS who are unable to walk or use walking devices would not benefit from the study as currently designed and they hope that future work in their area will include those who use wheelchairs and powerchairs.
- A question posed by the committee was how the principal investigator and healthcare providers will monitor for and manage overheating, nausea, and dizziness associated with increased physical activity.
- The committee wanted to highlight the importance of providing study information in a variety of ways to allow people with cognitive issues to participate.

▼ Project Information

Please confirm or update the project location where the project will take place.

**Project Location:**	Inside USA
**Project Country:**	United States
**Project State/Province:**	Washington
**Project City:**	Seattle
**Project Postal Code:**	98104
**Requested Start Date:**	10/1/2023
**Requested End Date:**	9/30/2026

Please check the ONE area that best represents your proposal.

General Discipline: Rehabilitation

You must select at least one keyword, however we encourage applicants to select 2-3 keywords that that best describe your proposal. For a full list of keywords, please click here.

Keywords List: Clinical Trials, Exercise/ physical activity, Rehabilitation

If a keyword is not in the list, you may add ONE keyword here:

Please check ALL categories that apply to your proposal.

Materials/Subjects: Human subjects, Clinical trial(s) data

Has this project been submitted to or will it be submitted to another agency for review? No

Would you consider yourself to be a new investigator?

New Investigator: No

▼ Principal Investigator and Institutional Official

For this proposal, please identify the Principal Investigator and Institutional Signatory.

The **Principal Investigator** should be you, the applicant. The individual selected in this field will receive all communications for this application. The PI is responsible for the application content, submitting full application to the signatory for authorization, and any resulting award.

The **Institutional Signatory** is the institutional official who has the legal authority to act and speak for the institution and is authorized to act on behalf of the institution and to assume the obligations. The Signatory will be responsible for authorizing the application in MSGrants to complete the application submission.

The **Additional Organization User fields** are *not required* but can be used to allow other contacts within your organization, such as a grant administrator or financial officer, access to view and edit your application. These individuals will be able to access your application at all stages, and if the application is approved they will see the grant record as well as see and submit grant reports; however, these individuals cannot submit an application to the signatory. These individuals will also be copied on automatic emails related to the progress of your application.

If a contact is not listed as an option, an account profile for the individual does not exist and a new account must be created in order to be listed.

**Organization**	University of Washington
**Organization Location**	Seattle ,WA
**Principal Investigator**	Dawn Ehde
**Institutional Signatory**	Carol Rhodes
**Additional Organization User (optional)**	
**Additional Organization User - Finance (optional)**	

Financial Officer

Please provide contact information for the institution's financial officer.

**First Name:**	Juan
**Last Name:**	Lepez
**Email:**	gcahelp@uw.edu
**Phone:**	206-616-9995

▼ Research and Project Team

List the names, affiliations, roles and responsibilities of all key personnel involved in this plan. Include any information that will help establish the competence of the research team to pursue the proposed plan.

Project Staff

Dawn | Ehde | University of Washington
Professor | Principal Investigator | — Edit | View

Kevin | Alschuler | University of Washington
Professor | Co-Investigator | — Edit | View

Cindy | Lin | University of Washington
Clinical Associate Professor | Co-Investigator | — Edit | View

Tracy | Herring | University of Washington
Acting Assistant Professor | Co-Investigator | — Edit | View

Sarah | Simmons | University of Washington
Assistant Professor | Co-Investigator | — Edit | View

Andrew | Humbert | University of Washington
Assistant Professor | Co-Investigator | — Edit | View

Laurie | Kavanagh | University of Washington
Research Manager | Other | — Edit | View

Susan | Robles | University of Washington
Research Study Coordinator | Other | — Edit | View

Otari | Ioseliani | University of Washington
Senior Software Engineer | Other | — Edit | View

Karla | Landis | University of Washington
Associate Director | Other | — Edit | View

Upload a biographical sketch in PDF format for all key personnel working on the project, including the PI. NIH format is preferred but you can download a template here. Press the green "+" button below to upload the biographical sketches. If you need to edit or delete a file, please click on the document title to display the edit tools.

Please select "Biosketch" via the drop down menu in the pop-up window when adding your file(s).

BIOGRAPHICAL SKETCHES

Biosketch_Ehde.pdf
Biosketch
Added by Dawn Ehde at 4:56 PM on January 23, 2023

Biosketch_Alschuler.pdf
Biosketch
Added by Dawn Ehde at 4:56 PM on January 23, 2023

Biosketch_Lin.pdf
Biosketch
Added by Dawn Ehde at 4:56 PM on January 23, 2023

Biosketch_Humbert.pdf
Biosketch
Added by Dawn Ehde at 4:56 PM on January 23, 2023

Biosketch_Herring.pdf
Biosketch
Added by Dawn Ehde at 4:56 PM on January 23, 2023

Biosketch_Simmons.pdf
Biosketch
Added by Dawn Ehde at 4:56 PM on January 23, 2023

▼ Scientific Summary of Proposed Project

Please provide a summary of the proposed research, including hypothesis, specific aims and how the proposal addresses the Society's Research Priorities for this RFA. Please limit your summary to 600 words or less. This summary does not need to include references or citations. Please note that any special characters or symbols that you enter here, such as Greek letters, will be converted to plain text. If Greek letters are used, they should be spelled out, e.g., "alpha", "beta", etc.

Scientific Summary :

Physical activity promotion is one of the most effective rehabilitation strategies for reducing multiple sclerosis (MS) symptoms and improving health and functioning. Despite the recognized benefits and the availability of guidelines for promoting physical activity, only 20% of adults with MS in the United States get sufficient physical activity. A major barrier to physical activity is that physical activity promotion is poorly integrated into MS healthcare. Although patients with MS report wanting information about exercise and activity prescriptions from their healthcare providers, most report that their providers rarely discuss, promote, or prescribe physical activity. Members of our UW Medicine team have been at the forefront of addressing this issue, developing Exercise Rx as an innovative approach to facilitating direct patient-provider interaction regarding physical activity promotion with the assistance of technology. Exercise Rx is an innovative software platform consisting of a patient app and a provider dashboard that is integrated into the electronic health record. Healthcare providers use the platform to prescribe exercise tailored to patients' ability levels and preferences. The platform includes behavior change strategies, including motivational feedback and nudges. It also facilitates a feedback loop with the healthcare provider, fostering patient-provider interactions around activity promotion and behavior change. Exercise Rx requires patients to only have their own smartphone, as it leverages existing phone capabilities to track, quantify, and tailor personalized physical activity solutions. Growing evidence suggests that Exercise Rx is effective in physical activity promotion in non-MS settings. However, its efficacy in improving physical activity engagement in people living with MS has not been tested.

This application proposes a randomized controlled trial designed to answer the question of whether Exercise Rx improves physical activity and other common symptoms and functional outcomes in adults with MS who are physically inactive (N = 106). Aim 1 proposed to evaluate the efficacy of Exercise Rx relative to usual care in increasing average daily step count (measure of physical activity: primary outcome) in adults with MS who are physically inactive. Aim 2 will evaluate the efficacy of Exercise Rx relative to usual care on secondary outcomes of importance to people with MS, specifically symptoms (fatigue, pain, and depressive symptoms), and functional outcomes (fatigue interference, pain interference, exercise self-efficacy). Outcomes will be collected at baseline, post a 12-week treatment period (primary endpoint), and at a 3-month follow up. Secondary analyses will test maintenance of any treatment effects at 3-months. In exploratory analyses, we will explore moderators of immediate and 3-month outcomes that could be evaluated a priori in future effectiveness and mechanism research. Aim 3 will explore implementation factors, including treatment adherence, patient/provider satisfaction, and barriers/facilitators (of physical activity as well as Exercise Rx implementation in clinical settings) relevant to future effectiveness trials and large-scale implementation of Exercise Rx in individuals with MS and their providers.

▼ Plain Language Description

The plain language description should describe your proposed research in language suitable for a news release to the lay public. This is important information for the society, and we please ask that applicants do not repurpose a scientific abstract for the plain language description. The plain language description is broken into several sections. In each section you are required to address a different element of your proposed project. Please read these instructions carefully and ensure that you address each element.

Applicants are advised to utilize the many resources the Society had provided on how to write a successful Plain Language Description found here (.pdf). If your project is funded, Society staff may make editorial changes to the summary to enhance its clarity.

Note: All research proposals deemed to be of scientific merit through the scientific peer review process undergo a Community Review to help us identify participant-centered concerns about proposed research, such as unduly burdensome protocols or outcomes of low interest to people affected by MS. These insights, along with the outcomes from the peer review, will be taken together to inform the final funding recommendations. The Community Review Committee will rely heavily on the Plain Language Description for each proposal.

What is the problem related to multiple sclerosis that you are addressing with this project? Please share your overall hypothesis(es) in plain language.

Promoting physical activity is one of the most effective rehabilitation strategies for reducing multiple sclerosis (MS) symptoms and restoring health and function. Regular physical activity improves mobility, balance, fatigue, and other symptoms in people with MS. Increased physical activity may also have disease-modifying effects. However, only 20% of adults with MS get the recommended amount of weekly physical activity. People with MS would like information about increasing their activity and prefer to hear directly from a provider with expertise in MS, rather than by receiving educational handouts or online information. MS healthcare providers also want to promote physical activity in their patients with MS, yet they lack tools and strategies to help their patients start, track, and maintain physical activity. Research has shown that support and guidance from healthcare providers, as well as behavior change strategies such as tracking of physical activity, setting appropriate goals, having an accountability partner, and using self-management skills to minimize potentially interfering symptoms such as fatigue (e.g., activity pacing) facilitate being active.

In response to this issue, our team at UW Medicine (University of Washington, Seattle, WA) developed ExerciseRx, a new, innovative approach to physical activity promotion that has been used in other medical clinics. ExerciseRx was created for healthcare providers to prescribe and monitor physical activity as part of routine care. ExerciseRx helps patients and healthcare providers communicate about ways to get more physical activity through use of an app on a patient's personal smartphone and the electronic health record. Healthcare providers use the easy-to-use ExerciseRx platform to prescribe physical activity tailored to patients' ability levels and preferences. It facilitates getting feedback from and interacting with the provider, while also serving as an easy-to-use, free tool to help patients and providers with goal setting, accountability, and activity tracking.

How do you propose to address the problem? Please summarize your aims using plain language.

The primary objective of this study is to test the effectiveness of ExerciseRx in increasing physical activity in adults with MS who are insufficiently active (defined as fewer than 150 minutes of physical activity per week). Physical activity will be measured by average daily step count and the volume of activity (a combination of how often one exercises and the intensity of exercise) over a period of 12 weeks. Our primary hypothesis is that participants randomized to ExerciseRx will show a greater increase in physical activity at 12-weeks, as measured by daily average step count (measured by participants' smartphone accelerometers), relative to those who are assigned to the standard care provided to them. Secondly, we will examine the effectiveness of ExerciseRx in improving symptoms important to people with MS, including symptoms of fatigue, pain, and depression. We will also examine other patient-reported functional outcomes, including the ability to participate in desired social roles and activities, and how much fatigue and pain interfere with what's important, as well as belief in one's ability to reach exercise goals. Finally, the study will also explore the adaptiveness of ExerciseRx for real world settings to better understand situational factors important to future use of ExerciseRx in MS healthcare settings. Some of the factors that will be assessed include treatment adherence (whether patients and providers used ExerciseRx), ease of use, what worked/what didn't, patient/provider satisfaction with ExerciseRx, and barriers/facilitators of physical activity during the study.

Describe the steps that you are taking to achieve these aims. Please be specific about the experiments you are conducting, and whether studies involve humans (with cohort details such as age, race, gender, etc.), models of MS, or cell cultures (human or animal).

We will conduct a randomized controlled trial in which adults with MS will be randomly assigned to either ExerciseRx or usual care to answer the question of whether ExerciseRx improves physical activity and other common symptoms (fatigue, pain, and depression) and functional outcomes (for example, participation in social roles and activities) in adults with MS who are insufficiently active. We plan to recruit 106 patients with MS who are insufficiently active from the University of Washington MS Center. We will also conduct qualitative interviews of approximately 15 participants who get ExerciseRx and their providers to get their perspectives on the ExerciseRx platform, such as ease of use, helpfulness, and how to improve it in future trials or use in MS care.

For studies that include people, please describe what is involved for participants in this study (number, frequency, and timing of visits; physical examinations; clinical measures; sample retrieval; imaging scans; symptom recording; etc.).

All participants will complete surveys at the beginning of the study (baseline), after the 12-week treatment period and at a 3-month follow-up.

Participants assigned to ExerciseRx will track their physical activity for 12 weeks. They will meet with their MS provider to develop a physical activity prescription tailored to their existing in physical activity habits and needs and eventually working up to meeting the recommendations, of at least 150 min of physical activity per week and gradually increasing activity goals based on individual abilities, preferences, and resources. To get their baseline step count, participants will carry their phone as they normally would for one week with no app or provider feedback; eligible iPhone and Android phones automatically track step count, which will provide the baseline data. Participants will then use the app daily for 12 weeks after the baseline week. Participants will receive a daily ExerciseRx app notification providing them with a personalized daily step target and weekly goal based on their baseline activity level. They will complete surveys about their barriers to being active and physical activity level, which are reviewed by their provider on the dashboard. Providers will monitor their dashboard weekly and they will offer feedback, guidance, and reinforcement via ExerciseRx.

Participants in usual care will receive the care they normally would receive at the MS Center, including encouragement to increase physical activity as tolerated, but without the use of ExerciseRx tools. After the 12-week intervention period, usual care participants will be offered the option of receiving ExerciseRx.

The qualitative interviews will allow us to understand patients' and providers' perspectives on ExerciseRx (e.g., what worked/didn't, barriers/facilitators), how an intervention may need to be adapted going forward, and, importantly, what is needed to support patients, providers, and healthcare systems to implement ExerciseRx in the real world in the future. This will consist of one-on-one interviews via Zoom.

How might the results of this study potentially make life better for people affected by multiple sclerosis? Please include a time frame.

Consistent with the National Ms Society's Pathways to Cure Restore pathway, the proposed rehabilitation research study aims to restore lost function by reversing damage and symptoms via increased physical activity, which is known to improve function, reverse symptoms, and enhance quality of life in adults with MS. If the study's results show that ExerciseRx improved physical activity, MS symptoms, and/or function in those who got it relative to those who did not, it would provide important scientific evidence for using ExerciseRx in the future. For example, if at the end of the 3-year study the results show that ExerciseRx is beneficial in increasing physical activity, such findings would indicate that ExerciseRx is another tool to help people living with MS restore lost function and improve quality of life. It may also help MS providers support their patient's wellness goals and build on the MS Society's wellness initiatives, which recognize that wellness strategies, including exercise, are priorities for people with MS. In addition, given the project's design to explore implementation experiences, if effective this could potentially help plan for the next stage of research and accelerate the use of the ExerciseRx intervention in other MS clinics.

▼ Proposal Narrative

Upload your completed narrative in PDF format and please select "Proposal Narrative" from the drop down menu in the pop-up window when adding your file(s). If you need to edit or delete a file, please click on the document title to display the edit tools.

PROPOSAL NARRATIVE

Proposal_Narrative.pdf
Proposal Narrative
Added by Dawn Ehde at 3:31 PM on February 3, 2023

▼ Letters of Support/Collaboration

This is the section where letters submitted with your application are uploaded, such as letters of collaboration or support from any individuals whose cooperation is necessary for the proposed research.

Letters must be converted to PDF format and should be combined and should be uploaded as a single PDF file. Please select "Letters" from the drop down menu in the pop-up window when adding your file(s).

LETTERS SUBMITTED

Letters_of_Support.pdf
Letters
Added by Dawn Ehde at 11:55 AM on February 3, 2023

▼ Materials Submitted

This is the section where appendix materials submitted with your application are uploaded. Appendix materials must be in PDF format and can be uploaded as either a combined file or multiple PDF files. Do not use this section to circumvent the page limits of the narrative section; putting information here that belongs in the narrative may result in a negative assessment by the peer review panel.

Items commonly included in the appendix are:

- Publications—Reprints, preprints, manuscripts, etc. that are relevant to the proposed project can be included as part of the appendix.
- Clinical Trial Protocols—In some cases, a proposal submitted to the Society may involve a study that is an add-on to a clinical trial that has been funded by another agency. Although the clinical trial itself is not the focus of the proposal, it is essential for the peer-review panel to have detailed information about the trial. If the above scenario describes your proposal, you must include a copy of the clinical trial protocol as part of the appendix.
- Other Materials—If your proposed study utilizes questionnaires or other types of printed assessment instruments, you may want to include these as part of the appendix. Many other types of materials could potentially be included in the appendix. However, keep in mind that reviewers have a great deal of material to review and so you should choose your appended materials carefully.

Please select "Materials" from the drop down menu in the pop-up window when adding your file(s). If you need to edit or delete a file, please click on the document title to display the edit tools.

MATERIALS SUBMITTED

▼ Human Subjects and/or Vertebrate Animals

Instructions

The applicant institution has the primary responsibility for protecting the rights and welfare of human subjects and for ensuring the humane care and use of animals in all research activities supported by the National Multiple Sclerosis Society, and of informing the Society of all relevant assurances and certifications. If an award is made as a result of this application, it is the responsibility of the grantee or fellow and the institution to inform the Society within a reasonable time of any change in the research protocol.

By virtue of the signature of an official authorized to sign for the institution on this application, the institution is declaring that all applicable Federal, State and Local regulations will be followed during the tenure of any grant awarded as a result of this Application. This form must be completed and submitted with any application to the Society for the support of research or training. In addition, a copy of the approval letter(s) signed by the Chairperson of the Institutional Review Board (IRB) and/or the Institutional Animal Use and Care Committee, as appropriate, must accompany any application. No funds will be dispersed for any award until these materials have been received, reviewed and accepted by the Society.

Will Human Subjects Be Used? Yes

Institutions in the United States:

If the IRB review is pending, approval must be obtained and evidence of approval submitted to the Society prior to funding. If IRB approval has been granted, a copy of the approval letter must be submitted as part of the PDF file that is uploaded in the Documents section.

Please select "Human Subjects and/or Vertebrate Animals" from the drop down menu in the pop-up window when adding your file(s).

**IRB Approval :**	Pending
**If Exempt, Provide Reason:**	
**Approval Date:**	
**Expiration Date:**	
**IRB Protocol Number:**	

The "Assurance of Compliance" Number issued to the applicant institution by the Federal Office of Protection from Research Risks: xc549863.

Assurance of Compliance Number: FWA00006878

Institutions Outside the United States:

Provide a description of the regulations applicable in your country, and evidence that such regulations will be followed for this project. You must submit either an assurance/certification number from the appropriate national agency or a statement signed by an official of your institution certifying that adequate protection of human subjects will be provided.

Please select "Human Subjects and/or Vertebrate Animals" from the drop down menu in the pop-up window when adding your file(s).

Foreign Institutions:

IRB APPROVAL FILE

Approval_STUDY00018628_Ehde_Initial.pdf
Human Subjects and/or Vertebrate Animals
Added by Phyllis Motta at 8:41 AM on August 31, 2023

Will vertebrate animals be used? No

▼ Budget

The **budget limit for 2023 is $660,000 in total direct costs for up to 3 years** of support.

Please confirm or update the total amount requested and indicate whether your budget will include subcontractor expenses.

Amount Requested (USD): 659,501.00

Will this project include subcontractors? No

To add a line item to the budget, please click on the "+" button below to open the form. The system will calculate the total budget per year after each line item has been added to the application. Please note, it is recommended that you save the application after each budget section has been completed to expedite the calculation process.

Personnel

Dawn Ehde | Principal Investigator Total Cost — View
Year 1: 39,482.00 | Year 2: 39,482.00 | Year 3: 39,482.00 | Year 4: | Year 5: |
Total: 118,446.00

Kevin Alschuler | Co-Investigator Total Cost — View
Year 1: 17,220.00 | Year 2: 17,220.00 | Year 3: 17,220.00 | Year 4: | Year 5: |
Total: 51,660.00

Tracy Herring | Co-Investigator Total Cost — View
Year 1: 10,709.00 | Year 2: 7,139.00 | Year 3: 7,139.00 | Year 4: | Year 5: |
Total: 24,987.00

Andrew Humbert | Co-Investigator Total Cost — View
Year 1: 7,820.00 | Year 2: 7,820.00 | Year 3: 23,459.00 | Year 4: | Year 5: |
Total: 39,099.00

Cindy Lin | Co-Investigator Total Cost — View
Year 1: 26,322.00 | Year 2: 21,057.00 | Year 3: 21,057.00 | Year 4: | Year 5: |
Total: 68,436.00

Sarah Simmons | Co-Investigator Total Cost — View
Year 1: 19,163.00 | Year 2: 12,776.00 | Year 3: 12,776.00 | Year 4: | Year 5: |
Total: 44,715.00

Laurie Kavanagh | Research Manager Total Cost — View
Year 1: 13,987.00 | Year 2: 6,993.00 | Year 3: 6,993.00 | Year 4: | Year 5: |
Total: 27,973.00

Susan Robles | Research Study Coordinator Total Cost — View
Year 1: 52,441.00 | Year 2: 52,441.00 | Year 3: 48,945.00 | Year 4: | Year 5: |
Total: 153,827.00

Otari Ioseliani | Senior Software Engineer Total Cost — View
Year 1: 26,516.00 | Year 2: 13,258.00 | Year 3: 13,258.00 | Year 4: | Year 5: |
Total: 53,032.00

Karla Landis, MS | Associate Director, Sports Institute Total Cost — View
Year 1: 15,042.00 | Year 2: 15,042.00 | Year 3: 15,042.00 | Year 4: | Year 5: |
Total: 45,126.00

**Total Year 1 Personnel:**	228,702.00
**Total Year 2 Personnel:**	193,228.00
**Total Year 3 Personnel:**	205,371.00
**Total Personnel Costs:**	627,301.00

Patient Costs

**Total Year 1 Patient Costs:**	0.00
**Total Year 2 Patient Costs:**	0.00
**Total Year 3 Patient Costs:**	0.00
**Total Patient Costs:**	0.00

Permanent Equipment

**Total Year 1 Equipment:**	0.00
**Total Year 2 Equipment:**	0.00
**Total Year 3 Equipment:**	0.00
**Total Equipment Costs:**	0.00

Consumable Supplies

Research Supplies | — View
Year 1: 400.00 | Year 2: 400.00 | Year 3: 400.00 | Year 4: | Year 5: |
Total: 1,200.00

**Total Year 1 Consumable:**	400.00
**Total Year 2 Consumable:**	400.00
**Total Year 3 Consumable:**	400.00
**Total Consumable Costs:**	1,200.00

Travel

Present at MS Conferences | — View
Year 1: 3,000.00 | Year 2: 3,000.00 | Year 3: 3,000.00 | Year 4: | Year 5: |
Total: 9,000.00

**Total Year 1 Travel:**	3,000.00
**Total Year 2 Travel:**	3,000.00
**Total Year 3 Travel:**	3,000.00
**Total Travel Budget:**	9,000.00

Other Expenses

Publication Costs | — View
Year 1: | Year 2: 1,500.00 | Year 3: 1,000.00 | Year 4: | Year 5: |
Total: 2,500.00

Participant incentives | — View
Year 1: 6,000.00 | Year 2: 6,000.00 | Year 3: 6,000.00 | Year 4: | Year 5: |
Total: 18,000.00

Provider incentives | — View
Year 1: | Year 2: | Year 3: 1,500.00 | Year 4: | Year 5: |
Total: 1,500.00

**Total Year 1 Other:**	6,000.00
**Total Year 2 Other:**	7,500.00
**Total Year 3 Other:**	8,500.00
**Total Other Expenses:**	22,000.00

▼ Final Budget Calculations

The system will generate a complete budget including all direct costs as entered above, and add indirect costs based on the information provided in the sections above. Please adjust the amount requested at the top of the Budget section (if necessary) so that it is equal to the calculated total budget in the final calculations.

Main Budget

	Year 1	Year 2	Year 3	Total
Personnel	228,702.00	193,228.00	205,371.00	627,301.00
Patient Costs				
Permanent Equipment				
Consumable Supplies	400.00	400.00	400.00	1,200.00
Travel	3,000.00	3,000.00	3,000.00	9,000.00
Other Expenses	6,000.00	7,500.00	8,500.00	22,000.00
Total Direct Costs	238,102.00	204,128.00	217,271.00	659,501.00
Main Budget Indirect Costs*	23,810.20	20,412.80	21,727.10	65,950.10
All Subcontracts (Direct Costs)				
Subcontract Indirect Costs				
**Total**	261,912.20	224,540.80	238,998.10	725,451.10

(Indirect Costs = 10% of direct costs except for patient costs and equipment)

▼ Budget Justification

Please provide a justification for the proposed budget. If applicable, please also include the subcontractor budget justification in the space below.

Budget Justification:

Key Personnel

Dawn Ehde, Principal Investigator, (1.8 calendar months effort Years 1-3)

Dr. Ehde, a clinical psychologist, holds the Nancy and Buster Alvord Endowed Professorship in Multiple Sclerosis Research in the Department of Rehabilitation Medicine, UW Medicine. She is also co-director of the UW Multiple Sclerosis Rehabilitation and Wellness Research Center. Dr. Ehde has been the Principal Investigator or a Co-Investigator on numerous extramural research grants, including more than 20 randomized controlled trials evaluating the efficacy or comparative effectiveness of cognitive behavioral, mindfulness, physical activity, and pharmacological interventions for chronic pain, fatigue, and/or depression in adults with multiple sclerosis (MS) or other chronic neurologic conditions. Of importance to the current proposal, Dr. Ehde has led the development and implementation of novel behavioral and rehabilitation interventions that have been deployed via telemedicine and in the MS clinical setting. Thus, she has extensive experience in overseeing and implementing trials such as the one proposed.

As PI, Dr. Ehde will be responsible for the overall implementation of the study. She will: (1) lead the refinement of the study protocol, including the recruitment/enrollment, intervention, assessment, and participant tracking procedures; (2) ensure that the clinical trial is registered at ClinicalTrials.gov; (3) supervise and facilitate decisions concerning implementation of study procedures and data coding, entry and analysis; (4) supervise the grant budget; (5) chair weekly staff meetings, during which study progress, including participant recruitment, enrollment, and retention, will be monitored; (6) complete progress reports; (7) work closely with the co-investigators, including the biostatistician, Dr. Humbert, in making decisions concerning data analyses; and (12) lead dissemination of the study findings.

Kevin Alschuler, PhD, Co-Investigator, (0.9 calendar months effort Years 1-3)

Dr. Alschuler is a Professor in the Department of Rehabilitation Medicine and Adjunct Professor in the Department of Neurology at the UW Medicine. He is also an Attending Psychologist, Co-Medical Director, and Director of Rehabilitation Research in the UW Medicine Multiple Sclerosis Center. Dr. Alschuler co-directs the University of Washington Multiple Sclerosis Rehabilitation and Wellness Research Center with Dr. Ehde, where he has extensive experience in both observational and intervention research in the MS population. He has collaborated on over 20 grant funded projects, with a large proportion of his work leveraging wearable technology, telehealth, videoconference technology, and innovative healthcare models. The study will benefit from his expertise in MS clinic-based research and his leadership role as Co-Medical Director of the MS Center. In the proposed study he will work closely with Dr. Ehde and the rest of the study team to ensure the successful implementation of the Exercise Rx intervention and study in the clinic, including facilitating recruitment of study participants and integrating the study procedures, including Exercise Rx, into provider workflows. He will work closely with the investigators to assist with protocol development and execution, aid in the refinement of intervention materials, supervise study staff (he is the Research Manager's direct supervisor), serve as the primary liaison to the clinicians using Exercise Rx, and participate in the analysis and dissemination of study results.

Andrew Humbert, PhD, Co-Investigator, (0.6 calendar months effort Years 1 & 2, 1.8 calendar months Year 3)

Dr. Humbert is a biostatistician and Assistant Professor in the Department of Rehabilitation Medicine at UW Medicine. He has 9 years of experience working as a biostatistician with interdisciplinary research teams. He has collaborated with Drs. Ehde and Alschuler on several studies in the MS population. Dr Humbert has also collaborated with Dr. Lin, a co-investigator on the proposed study and developer of Exercise Rx, and her team; he has analyzed the role of physical activity on hospital utilization and data from an Exercise Rx pilot study. Thus, he is well-suited to overseeing and conducting the study's data analyses, having used Exercise Rx data previously as well as data from the UW Medicine electronic medical record system. He has the expertise to collaborate with the research team on the proposed grant assessing the impact of Exercise Rx on adults with multiple sclerosis. As the biostatistician, Dr. Humbert will: (1) assist the research team in finalizing the biostatistical design; (2) oversee the biostatistical design of the study, the structure of study databases, and data quality monitoring procedures; (3) conduct data analysis; (4) collaborate in the interpretation of the study results; and (5) collaborate in dissemination activities.

Cindy Lin, MD, Co-Investigator, (1.2 calendar months effort Years 1, 0.96 calendar months effort Years 2 and 3)

Dr. Lin, a board-certified physiatrist, is the Associate Director of Clinical Innovation at The Sports Institute at UW Medicine and Clinical Associate Professor in the Department of Rehabilitation Medicine at UW Medicine. Dr. Lin leads the Exercise Rx project, a collaborative effort between The Sports Institute, UW Computer Science & Engineering Ubicomp Lab, and UW Human Centered Design and Engineering. She will be responsible for providing oversight and leadership of Exercise Rx clinical research implementation as well as educating providers in the MS Center on how to use Exercise Rx, serving as a clinical resource to the MS providers, supervising software engineering needs and UI/UX design, and coordinating with the MS Center clinic. Given her experience in developing and testing Exercise Rx in other populations, she will also participate in the analysis and dissemination of study results. We request more FTE for her in Year 1 given that she will be involved in finalizing the Exercise Rx protocol for the MS providers and will train them in its use during the first year.

Sarah Simmons, MD, PhD, *Co-Investigator*, *(0.9 calendar months effort Year 1, 0.6 calendar months effort Years 2 & 3)*

Dr. Simmons is a physiatrist and Assistant Professor in the Department of Rehabilitation Medicine who obtained her medical degree, PhD (Immunology), and residency (Physical Medicine and Rehabilitation residency) at the UW School of Medicine. She recently completed a fellowship in Neuroimmunology and MS at the Cleveland Clinic Foundation, where she conducted research on exercise interventions, clinical trial design, MS symptom management, and clinical outcomes in the MS population. She also obtained advanced clinical training and experience in the rehabilitation management of adults with MS. As the physiatrist with MS expertise on the project, Dr. Simmons will assist the team in finalizing the protocol to ensure it is appropriate for the MS patient population, consult with the study team on questions about a potential participant's MS diagnosis and appropriateness for the study from a medical standpoint, attend weekly project staff meetings, and provide medical oversight of the protocol. As a physiatrist in the UW MS Center, she will also serve as one of the clinicians who implements Exercise Rx with patients randomized to receive Exercise Rx as study participants. She will also assist the investigators in making decisions concerning overall implementation of study procedures and in preparing manuscripts and posters/papers for presentation at scientific meetings. We request more FTE for her in Year 1 given that she will collaborate with Dr. Lin's team from the Sports Institute in finalizing the study protocols that will be used in this study so that they are relevant to the MS population and clinical setting from a medical standpoint.

Tracy Herring, PhD, *Co-Investigator*, *(0.9 calendar months effort Year 1, 0.6 calendar months effort Years 2 & 3)*

Dr. Herring is a rehabilitation psychologist and Acting Assistant Professor in the Department of Rehabilitation Medicine at UW Medicine. She is an attending psychologist at the UW Multiple Sclerosis Center, where she provides psychological services to adults living with MS, including helping them manage common co-morbid symptoms such as fatigue and chronic pain, and make lifestyle behavior changes, such as increasing physical activity. Her research interests include non-pharmacological management of symptoms and optimizing coping and adjustment in MS. Dr. Herring was part of the initial team that adapted Exercise Rx to the MS population; she was involved in beta testing it and refining the behavioral messages and prompts to be relevant to adults with MS. In this study she will assist the team in finalizing the protocol to ensure it is appropriate for the MS patient population from a behavioral standpoint. As a psychologist in the MS Center, she will also serve as one of the clinicians who implements Exercise Rx with patients randomized to receive Exercise Rx as study participants. She will also participate in the interpretation of the study results and dissemination activities. We request more FTE for her in Year 1 given that she will collaborate with Dr. Lin's team from the Sports Institute in finalizing the study protocols that will be used in this study so that they are relevant to the MS population and clinical setting from a behavior change standpoint.

Other Personnel

Laurie Kavanagh, MPH, Research Manager, (1.2 calendar months effort Year 1, 0.6 calendar months effort Years 2 & 3)

Ms. Kavanagh is the Research Manager for the UW Multiple Sclerosis Rehabilitation & Wellness Research Center, where she directs multiple large randomized clinical trials and observational studies for Drs. Alschuler and Ehde. She has considerable expertise in grants management, including the financial, regulatory, Human Subjects, scientific, and supervisory aspects. Ms. Kavanagh will devote her effort to: (1) monitoring participant recruitment and data collection efforts, including by tracking the progress of recruitment for the study, providing regular recruitment reports to the study investigators, and discussing recruitment strategies with staff and investigators; (2) facilitating communication and meetings amongst all study-related individuals as needed, including, but not limited to: investigators, research participants, clinical staff, research staff, the general public, and UW administrative personnel including administration at the MS Center; (3) assisting the investigators with finalizing the study protocols and operationalizing them for an overall Manual of Operations for the study; (4) obtaining and maintaining UW IRB approval; (5) overseeing the development and use of participant tracking and other reports used by the investigators to track the study's progress and complete reports for the sponsor; (6) assisting with the fiscal management of the grant including monitoring budget expenditures and making purchase recommendations; (7) registering the trial on ClinicalTrials.gov and keeping the trial registration up-to-date; and (8) supervise Susan Robles, the research study coordinator that will be assigned to this project.

Susan Robles, Research Study Coordinator *(9.0 calendar monthseffort Years1 & 2, 8.4 calendar months effort Year 3)*

Ms. Robles is a Research Study Coordinator for the UW Multiple Sclerosis Rehabilitation & Wellness Research Center, where she works closely with and under the supervision of Ms. Kavanagh to carry out the tasks associated with the Center's portfolio of research studies. Ms. Robles will devote her effort on this project to assisting Ms. Kavanagh on the tasks described above, including assisting with setting up study protocols and procedures,

including the Manual of Operations. Ms. Robles will also be responsible for (1) recruiting, enrolling, and retaining study participants, (2) facilitating data collection; (3) paying incentives to participants for their completed outcome assessments; and (4) generating weekly tracking reports of participant enrollment and retention rates. Ms. Robles will also engage in other tasks that support the study at Ms. Kavanagh's direction, such as with facilitating communication across study teams, monitoring study supplies and equipment and replace and/or update as necessary; and assisting with the UW IRB reports, and other miscellaneous study tasks. We have budgeted a slightly reduced FTE in Year 3, as her assistance will not be needed in the last two months of the study.

Otari Ioseliani, Senior Software Engineer (2.4 calendar months effort Year 1, 1.2 calendar months effort Years 2 & 3)

Otari Ioseliani is a Senior Software Engineer at the UW Computer Science & Engineering Ubicomp Lab. He has developed iOS, Android, and web applications with 25,000+ daily visitors for Washington State University and UW. Mr. Ioseliani is the software engineer who developed the Exercise Rx smartphone application and the provider dashboard at UW and will be responsible for all software engineering needs pertaining to the use of the Exercise Rx patient app and provider dashboard in the MS Clinic, as well as app-based data collection, storage, and reporting.

Karla Landis, MS, Associate Director, Sports Institute, (1.2 calendar months effort Years 1–3)

Ms. Landis is the Associate Director of The Sports Institute at UW Medicine, where she assists with the development and execution of its program and research strategy, staffing, community outreach and capacity building. Ms. Landis works closely with Dr. Lin to carry out tasks associated with expanded use of Exercise Rx through research. On this project, she will devote her effort to training and supporting the UW MS Center's research staff (Ms. Kavanagh and Robles) on tasks pertaining to onboarding staff and providers in the use of Exercise Rx, specifically the Exercise Rx patient app. Ms. Landis will also serve as project manager to liaise between the research and engineering teams, manage and communicate milestones pertaining to Exercise Rx, and problem-solve with the study team as needed.

Fringe Benefit Rates

The University of Washington applies the following fringe benefit rates in accordance with the current DHHS negotiated agreement for this proposal: faculty 24.1%, classified staff 39.5%, and professional staff 31.8%. Upon funding of this proposal, rates may adjust in accordance with current approved UW DHHS rates.

Equipment

None requested

Supplies

Research supplies

Total $1,200 ($400/year Years 1-3)

We anticipate incurring costs of $400/year in research supplies (e.g., paper, folders, pens, toner, etc.) over the course of the study.

Scientific Travel

Funding is requested each year for the PI (Dr. Ehde), Dr. Lin, and/or other investigators to attend an annual scientific conference, potentially the annual meetings of the Consortium of MS Centers or the American Congress of Rehabilitation Medicine, for purposes of presenting the study's protocol (year 1) and findings (years 2 and 3). We are requesting $3,000 per year for airfare, lodging, and per diem.

Other Expenses

Participant incentives ($6,000/year Years 1, 2, & 3)

Funds are requested to cover an incentive of $150 per participant (N = 106) for completion of outcome assessments ($50 each for baseline, post-treatment, and follow-up assessment points) and $150 per participant for up to 14 ExerciseRx participants to complete the semi-structured interviews. The total requested funds in this category are $18,000.

Provider incentives ($1,500 Year 3)

Funds are requested to cover an incentive of $500 for 3 providers to complete the digital health tool usability assessments and provide qualitative feedback on their experience using the Exercise Rx provider dashboard as part of patient care ($100 per hour, up to 15 hours of provider time).

Indirect Costs

The University of Washington applies F&A costs in accordance with negotiated agreement with the Department of Health and Human Services, dated 09/30/20 and preceded on 07/21/17. The approved F&A rate for sponsored research conducted on the UW Main Campus is 55.5% for the period July 1, 2018 onward. The sponsor limits indirect costs to 10%, however, so that rate is instead applied.

Subcontractor Budget Justification:

▼ Other Support

Press the green "+" button below to upload your current and pending support for the principal investigator and all other professional personnel listed in this application. Please select "Other Document" from the drop down menu in the pop-up window

Include both current and pending funding sources, NIH-style acceptable. Show the agency to which the application(s) was/were submitted (for NIH, include the institute and grant number) indicate the title of each project, its duration, and the percent effort of the individual. This listing should include support from all sources including governmental, non-profit, foundation, private philanthropic, and other sources. Briefly describe the specific aims of each project and how they relate to this project. List the support in US dollars for the current year and the total support in US dollars for all years.

A template is available here.

OTHER SUPPORT

Other_Support_Ehde.pdf
Other Document
Added by Dawn Ehde at 4:58 PM on January 23, 2023

Other_Support_Alschuer.pdf
Other Document
Added by Dawn Ehde at 4:58 PM on January 23, 2023

Other_Support_Lin.pdf
Other Document
Added by Dawn Ehde at 4:58 PM on January 23, 2023

Other_Support_Humbert.pdf
Other Document
Added by Dawn Ehde at 4:58 PM on January 23, 2023

Other_Support_Herring.pdf
Other Document
Added by Dawn Ehde at 4:58 PM on January 23, 2023

Other_Support_Simmons.pdf
Other Document
Added by Dawn Ehde at 4:58 PM on January 23, 2023

▼ Applicant Demographics

The Society is requesting this information to be used specifically to help inform the Society's Diversity, Equity, and Inclusion initiatives (please see our Cultural Values and Diversity, Equity, and Inclusion Statement online here). Your individual responses will be confidential and will not affect consideration of your application. Please note we may share aggregate and anonymized summaries externally with Society advisory boards as part of ongoing discussions on our funding programs and with broader Society constituents as part of our transparency efforts. For more information feel free to contact Society staff.

What is your race or ethnicity? White

What is your gender identity? Female

▼ Award Documentation

This award notice contains the key information concerning your award along with the Award Acceptance Form. Complete the Award Acceptance Form, have it signed by the appropriate institutional officer, convert it to PDF format, and upload it here.

Obtain a completed W-9 from your institution or you can download the W-9 form here. Complete the W-9, have it signed by the appropriate institutional officer, and upload it here. (This only applies to institutions in the U.S.)

Upload the appropriate signed IRB and/or animal protection approval memo(s) in PDF format here.

PAYMENT SCHEDULES: Please note, payments are generally made on a quarterly basis in accordance with the budget and contingent upon the Awardee fulfilling all applicable milestones and reporting obligations, unless otherwise stated in your agreement. For specific questions on your payment schedule please reach out to your grant administrator: **Phyllis Motta, phyllis.motta@nmss.org**.

FULLY EXECUTED AGREEMENT - NMSS STAFF UPLOAD ONLY

NMSS_Countersigned_Ehde_RFA.pdf
Fully Executed Grant Agreement
Added by Phyllis Motta at 6:23 AM on October 16, 2023

NMSS_Countersign RFA_Ehde.html
Fully Executed Grant Agreement
Added by Phyllis Motta at 6:42 AM on August 31, 2023

AWARD AMENDMENTS

AWARD DOCUMENTS AND APPROVED BUDGET

OSP signed_Ehde_RFA Notification of Award.pdf
Fully Executed Grant Agreement
Added by Dawn Ehde at 1:47 PM on August 29, 2023

ACH and WIRE instructions for Main UW 5000 1.25.2023.pdf

Added by Carol Rhodes at 9:55 AM on August 29, 2023

Ehde-RFA Approved Budget.pdf.pdf
Approved Budget
Added by Phyllis Motta at 10:52 AM on August 2, 2023

Ehde_RFA Notification of Award.pdf
Award Document
Added by Phyllis Motta at 10:52 AM on August 2, 2023
